# Histoplasmosis in a fingolimod-treated patient: case report and scoping review

**DOI:** 10.1590/S1678-9946202466039

**Published:** 2024-07-08

**Authors:** Vítor Falcão de Oliveira, Guilherme Diogo da Silva, Larissa Teixeira Silva, Victor Lucas Gonçalves, Paula Emilia Rivas, Alexandre Coelho Marques, Mariane Taborda, Adriana Satie Gonçalves Kono Magri, Samira Luisa Apóstolos-Pereira, Dagoberto Callegaro, Marcello Mihailenko Chaves Magri

**Affiliations:** 1 Universidade de São Paulo, Faculdade de Medicina, Hospital das Clínicas, Departamento de Moléstias Infecciosas e Parasitárias, São Paulo, São Paulo, Brazil; 2 Universidade de São Paulo, Faculdade de Medicina, Hospital das Clínicas Departamento de Neurologia, São Paulo, São Paulo, Brazil; 3 Universidade de São Paulo, Faculdade de Medicina, Hospital das Clínicas Departamento de Patologia, São Paulo, São Paulo, Brazil

**Keywords:** Histoplasmosis, Fingolimod, Multiple sclerosis, Spingoshine-1-phosphate receptor

## Abstract

Fingolimod is a sphingosine-1-phosphate receptor modulator used to treat multiple sclerosis. While fingolimod has been associated with an increased risk of cryptococcal meningitis, its correlation with other deep mycoses remains unclear. In this study, we conducted a scoping review of fingolimod associated with histoplasmosis, based on a case report, a literature review, and data from the FDA Adverse Events Reporting System (FAERS) as of January 24^th^, 2023. A 30-year-old Brazilian woman diagnosed with relapsing-remitting multiple sclerosis, receiving a daily dose of 0.5 mg of fingolimod, presented with a two-month history of fever and unintended weight loss, accompanied by lymphadenopathy, splenomegaly, and lung involvement was investigated. Biopsy of a lung nodule revealed fungal structures suggestive of *Histoplasma sp.* Additionally, serological testing yielded positive for *Histoplasma capsulatum.* Disseminated histoplasmosis should be considered in the differential diagnosis of febrile syndromes in patients undergoing fingolimod therapy for multiple sclerosis, particularly in the Americas, where this mycosis is endemic. Treatment with itraconazole and modification of immunotherapy can achieve excellent clinical outcomes.

## INTRODUCTION

Fingolimod is an oral immunosuppressant that acts by binding to sphingosine-1-phosphate (SP) receptors, mainly used in the treatment of multiple sclerosis^1^. Modulation of SP receptors limits the egression of autoreactive lymphocytes from lymph nodes to peripheral circulation^2^. Thus, SP receptor modulation reduces disease activity in multiple sclerosis. However, this mechanism of action predisposes patients to viral and fungal infections^3^.

Fingolimod is associated with fungal infection by *Cryptococcus* spp.^4^, as described in the drug package insert. In addition to limiting lymphocyte egress from lymph nodes, modulation of the sphingosine-1-phosphate (SP) receptor can disrupt macrophages within granulomas, potentially explaining the reactivation of cryptococcosis^5^. Other granulomatous diseases, such as tuberculosis, have also been reported in patients receiving fingolimod treatment^6,7^. While a similar mechanism may contribute to other fungal infections, there is limited understanding of the association between fingolimod and additional deep mycoses. Our objective was to report a case of disseminated histoplasmosis in a patient using fingolimod. With more than 808,900 patient/year exposed to fingolimod in individuals with multiple sclerosis^8^, there is a pressing need for recognition and early treatment of this complication associated with SP receptor modulation therapy.

## CASE REPORT

This articles investigates the case of a 30-year-old Brazilian woman diagnosed with relapsing-remitting multiple sclerosis, receiving a daily dose of 0.5 mg of fingolimod, presented with a two-month history of fever and unintended weight loss. Common risk factors that lead to exposure to *Histoplasma* sp. as debris from old buildings, chicken coops, bird roosts, wood piles, and bat feces (cave exploration) were not identified. She had been using fingolimod for the past year, and her lymphocyte count was within the normal range (1.17 × 109/L). Laboratory testing revealed elevated levels of aspartate aminotransferase (180 U/L; normal < 31 U/L), alanine aminotransferase (264 U/L; normal < 31 U/L), alkaline phosphatase (327 U/L; reference range 35–104 U/L), gamma-glutamyl transferase (120 U/L; reference range 5–36 U/L), total bilirubin (0.93 mg/dL; reference range 0.2–1 mg/dL), and C-reactive protein level (7.2 mg/dL; normal < 0.5 mg/dL). Despite these aforementioned figure, no evidence of pancytopenia was found.

**Figure 1:**
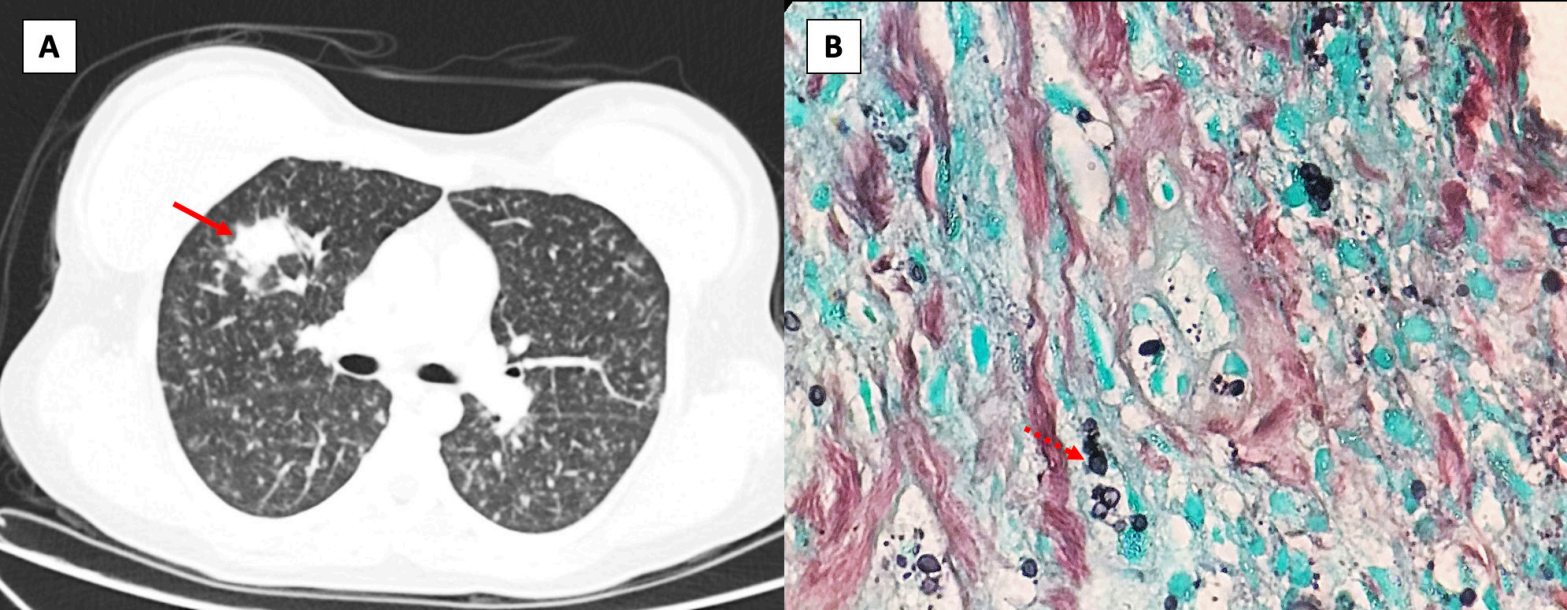
Histoplasmoma: A) Chest computed tomography showed a diffuse micronodular lung nodule in the right upper lobe (solid arrow); B) A Grocott-Gomori methenamine silver (GMS) stain highlighted yeast forms consistent with *Histoplasma* sp.

Abdominal computed tomography revealed splenomegaly with small sparse hypoattenuating foci associated with retroperitoneal lymphadenopathy. Chest computed tomography scan showed micronodular disease, mediastinal lymphadenopathy, and a lung nodule (Figure 1A). Disseminated tuberculosis was the initial hypothesis, however, bronchoalveolar lavage fluid microscopy, culture, and nucleic acid amplification tests yielded negative results. Biopsy of the lung nodule demonstrated granulomatous chronic inflammation with necrosis and fungal structures suggestive of *Histoplasma* sp. (Figure 1B). Additionally, serum immunodiffusion was positive for *Histoplasma capsulatum*, with counterimmunoelectrophoresis (CIE) titers of 1/128.

Treatment with itraconazole 400mg per day yielded excellent clinical, radiological, and serological improvement. Itraconazole was discontinued after 31 months of treatment, following normalization of CIE results. The disease-modifying drug was switched from fingolimod to natalizumab (anti-integrin therapy).

## DISCUSSION

We reported a case of disseminated histoplasmosis, which was presented with febrile syndrome associated with unintended weight loss, lymphadenopathy, splenomegaly, and lung involvement in a patient taking fingolimod for the treatment of multiple sclerosis. Diagnosis of the fungal infection led to appropriate treatment, with complete symptoms improvement and modification of immunotherapy.

We searched for case reports in PubMed for literature review. Our search terms were “Histoplasmosis” and “Fingolimod,” without any restriction regarding language or publication date. We only found two case reports^9,10^, which are summarized in Table 1.

**Table 1:** Summary of histoplasmosis case reports in patients using fingolimod.

Article	Year	Country	Age (years)	Sex	Symptoms	Sites of infection	Diagnostic method	Epidemiology	WBC count (× 10^9^/L)	Lymphopenia	Time of fingolimod treatment (months)	Antifungal therapy	Time of antifungal therapy (months)	Death
Abrahamowicz *et al*.^9^	2021	USA	46	Male	Fevers, night sweats, and weight loss	Liver and intestine	Histopathology and molecular testing	Contact with dead bats and rodents.	4.3	NA	36	Amphotericin followed by Itraconazole	NA	No
Veillet-Lemay *et al*.^10^	2017	Canada	54	Female	Plaques with central erosion and crusting	Skin	Histopathology and culture of biopsy	NA	NA	NA	36	Itraconazole	6	No

NA = Not available; WBC = white blood cell

Furthermore, we searched the public FDA Adverse Events Reporting System (FAERS) for additional cases of histoplasmosis on March 4^th^, 2024. Our search strategy included “Fingolimod hydrochloride,” “Fingolimod,” or “Gilenya” in Drugs section without date restriction, and “Histoplasmosis,” “Histoplasmosis Cutaneous,” “Presumed Ocular Histoplasmosis Syndrome,” or “Histoplasmosis Disseminated” in the Reaction section. We found 84,805 adverse events from 2008 to 2023, of which 14 non-published cases of histoplasmosis.

All cases occurred in the Americas, where *Histoplasma* spp. is endemic. Other systemic involvement of histoplasmosis included cutaneous lesions, hepatomegaly, and gastrointestinal symptoms. For cases describing patients’ sex, 50% of cases were female (4/8), with a median age of 51.5 years (IQR 43 - 56.3). Notably, only two deaths (14%, 2/14) were reported.

There is limited understanding regarding the risk factors for fungal infections in patients using fingolimod. Our patient had only been using the medication for one year and had normal lymphocyte counts, suggesting that the duration of therapy and lymphocyte counts might not serve as reliable predictors of fungal infection. Unfortunately, the other two case reports^9,10^ did not include information on lymphocyte counts. We speculate that fingolimod-induced disorganization of the lung histoplasmoma led to disseminated histoplasmosis, a phenomenon that may not be reflected by blood lymphocyte counts.

This case report suggests the importance of recognizing that fingolimod treatment may potentially predispose individuals to various granulomatous conditions beyond histoplasmosis, including tuberculosis^6,7^. This broader perspective is crucial for clinicians to consider when evaluating patients on fingolimod therapy, as it expands the differential diagnosis and ensures comprehensive management of potential treatment-associated complications. Overall, this report effectively highlights the need for vigilance regarding granulomatous diseases in patients receiving fingolimod.

Case reports can be useful to demonstrate a rare adverse effect of a drug. To reinforce this association, we performed a literature review that yielded only two more cases. Moreover, we also consulted the FAERS database, which showed more cases of histoplasmosis in participants using fingolimod. However, FAERS database analysis is limited by the lack of data about detailed clinical, laboratory, and treatment characteristics.

## CONCLUSION

Febrile syndromes in patients using fingolimod should include disseminated histoplasmosis in the differential diagnosis, a potentially fatal endemic mycosis in the Americas. Serum immunological tests such as immunodiffusion and counterimmunoelectrophoresis, or biopsy of visceral involvement of disease can diagnose this mycosis. Treatment with antifungal therapy and modification of immunotherapy can achieve excellent clinical outcomes.
